# Feasibility of implementing a surgical patient safety checklist: prospective cross-sectional evaluation

**DOI:** 10.1186/s40814-023-01277-3

**Published:** 2023-03-27

**Authors:** Kristin Harris, Eirik Søfteland, Asgjerd Litleré Moi, Stig Harthug, Mette Ravnøy, Anette Storesund, Elaheh Jurmy, Eli Skeie, Hilde Valen Wæhle, Nick Sevdalis, Arvid Steinar Haugen

**Affiliations:** 1 Department of Anesthesia and Intensive Care, Haukeland University Hospital, Bergen, Norway; 2 Department of Health and Caring Sciences, Western Norway University of Applied Sciences, Bergen, Norway; 3 Department of Clinical Medicine, University of Bergen, Bergen, Norway; 4 Department of Research and Development, Haukeland University Hospital, Bergen, Norway; 5 Department of Clinical Science, University of Bergen, Bergen, Norway; 6 Department of Surgery, Førde Central Hospital, Førde, Norway; 7 Faculty of Health Sciences, Centre for Resilience in Healthcare (SHARE), University of Stavanger, Stavanger, Norway; 8 Health Service & Population Research Department, Centre for Implementation Science, King’s College London, London, UK; 9 Department of Nursing and Health Promotion Acute and Critical Illness, Faculty of Health Sciences, OsloMet — Oslo Metropolitan University, Oslo, Norway

**Keywords:** Surgery, Checklist, Patient safety, Patient’s surgical safety checklist, Patient involvement

## Abstract

**Background:**

The World Health Organization’s Global Patient Safety Action Plan 2021–2030 call for attention to patient and family involvement to reduce preventable patient harm. Existing evidence indicates that patients’ involvement in their own safety has positive effects on reducing hospitalisation time and readmissions. One intervention reported in the literature is the use of checklists designed for patients’ completion. Studies on such checklists are small scale, but they are linked to reduction in length of hospital stay and readmissions. We have previously developed and validated a two-part surgical patient safety checklist (PASC). This study aims to investigate the feasibility of the PASC usage and implementation prior to its use in a large-scale clinical trial.

**Methods:**

This is a prospective cross-sectional feasibility study, set up as part of the design of a larger stepped-wedge cluster randomised controlled trial (SW-CRCT). Descriptive statistics were used to investigate patient demographics, reasons for not completing the PASC and percentage of PASC item usage. Qualitative patient interviews were used to identify barriers and drivers for implementation. Interview was analysed through content analysis.

**Results:**

Out of 428 recruited patients, 50.2% (215/428) used both parts of PASC. A total of 24.1% (103/428) of the patients did not use it at all due to surgical or COVID-19-related cancellations. A total of 19.9% (85/428) did not consent to participate, 5.1% (22/428) lost the checklist and 0.7% (3/428) of the patients died during the study. A total of 86.5% (186/215) patients used ≥ 80% of the checklist items. Barriers and drivers for PASC implementation were grouped into the following categories: Time frame for completing the checklist, patient safety checklist design, impetus to communicate with healthcare professionals and support throughout the surgical pathway.

**Conclusions:**

Elective surgical patients were willing and able to use PASC. The study further revealed a set of barriers and drivers to the implementation. A large-scale definitive clinical-implementation hybrid trial is being launched to ascertain the clinical effectiveness and scalability of PASC in improving surgical patient safety.

**Trial registration:**

Clinicaltrials.gov: NCT03105713. Registered 10.04.2017

**Supplementary Information:**

The online version contains supplementary material available at 10.1186/s40814-023-01277-3.

## Key messages regarding feasibility

- What uncertainties existed regarding the feasibility? Prior to conducting this feasibility study, few studies had been performed on patient checklist usage, and it was unclear whether patients were willing to use such a checklist and what the barriers and drivers would be.
- What are the key feasibility findings? Four out of five surgical patients who agreed to use the checklist completed ≥ 80% of the checklist items. However, there were some barriers identified related to surgical cancellations, recruitment and time frame for completion and design of the checklist that has to be addressed to facilitate its future use in research and clinical settings.
- What are the implications of the feasibility findings for the design of the main study?

- This study demonstrated that implementation of a patient-completed checklist to increase patient involved in their own safety is feasible. Based on the findings, it is clear that to ensure success, there is a need to provide adequate research funding, to allow employment of more research staff and to increase the numbers of clusters and recruitment time for a definitive trial of the checklist.

## Background

The World Health Organization’s Global Patient Safety Action Plan 2021–2030 emphasise the involvement of patients and family in safety as an essential strategy to reduce preventable patient harm [1]. A number of patient decision aids and prehabilitation programmes exist, indicating positive effects of patient involvement in their own treatment across a range of health specialities [2, 3]. Mobile applications have also been developed, e.g. addressing the surgical pathway (My Surgery app) or supporting cancer patients’ treatment [4, 5].

Checklists for patients to use themselves have recently started to appear in the literature. Only a few studies to date have investigated such checklists and their effects on patient outcomes. Hardiman and colleagues developed a patient checklist for postsurgical care for patients who had undergone ileostomy surgery [6]. Other patient checklists have been developed to aid discharge from hospital following medical admissions and one preoperative visit checklist aiming to increase parents’ understanding of the surgical consent process for paediatric orthopaedic patients [7, 8]. These studies suggest that patient checklists and similar interventions are feasible to use [6–8]. To our knowledge, only two studies to date have investigated patient-completed checklists’ feasibility and effectiveness. One study found a reduction in the rate of readmissions [6], while the other one demonstrated a reduction in overnight hospital stays [4]. Both studies had small numbers of participants and were single-centre studies. Furthermore, Russ and colleagues performed a multicentre study investigating the feasibility of the “My Surgery” surgical app. They found the app was feasible and empowered patients to be more involved in conversations on their own care. It also increased awareness of surgical risks and safety related to their behaviours [9].

More recently, our group developed a novel surgical patient safety checklist (PASC) [10]. This checklist has been developed over the past 5 years following recommended guidelines for patient checklist design [11] and with the main aim to increase patients’ involvement in the safety of their own care [10, 12]. To date, we have reported on development and content validity of PASC, and we have optimised its usability through a reduction in number of items and clarifications of checklist content [10].

Larger studies, with a range of clinical and implementation outcomes, are needed to establish robustly whether checklists aimed for use by patients can be applied widely and whether doing so improves the outcomes and experiences of patient care. To address this need, we have designed a stepped-wedge cluster randomised controlled trial (SW-CRCT) on PASC (ClinicalTrials.gov: NCT03105713). To identify PASC usage, recruitment and barriers and drivers to implementation prior to the large-scale SW-CRCT, we have designed a feasibility study. The aim was to assess PASC feasibility regarding recruitment and from a user perspective that can affect the planned SW-CRCT.

## Method

This study is a feasibility study with a prospective cross-sectional evaluation design, set up as part of the SW-CRCT on PASC (see above). To investigate patients’ acceptance for PASC and their barriers and drivers to using PASC, focus-group interviews were also carried out in this feasibility study. PASC consists of two parts, one preoperative and one postoperative set of checks. The preoperative checks (30-item checks and 2 advisory items) cover medical and medication history, optimisation of own health and important information and preparations before surgery. The postoperative checks (26-item checks) cover complications, physical activity and restrictions, medication safety and further treatments and follow-ups after surgery. Both parts of PASC are provided to the patients in paper format 2 to 12 weeks before surgery. The content and wording of the PASC items are based on extensive developmental and validation work with patients and healthcare professionals, reported in a previous study [10]. The overall PASC content was reduced to 27 preoperative items and 20 postoperative items based on the data collection in the aforementioned study. The full version of the checklist was translated to English and published [10]. Please see additional file 1 for PASC item questions and for the item response rate. The SW-CRCT is planned to be carried out at the same clinical settings as this feasibility study. Elective surgical patients in the control group will receive care as usual before and after surgery, while elective surgical patients in the intervention group will receive both parts of PASC on paper and electronically two to twelve before surgery. Based on the power calculation performed after the completion of this feasibility study, we plan to include 5320 elective surgical patients in the SW-CRCT. The recruitment will be carried out in seven different clusters over a total period of 20 months. The primary outcome measures will be complications, mortality up to 30 days postoperatively and length of stay (LOS).

To investigate PASC’s feasibility, elective surgical patients were asked to use both parts of the checklist (see sections below). The CONSORT extension for feasibility studies and Reporting Qualitative Research (COREQ) checklist was used to report this study [13, 14].

### Setting and participants

Elective surgical patients were recruited from two Norwegian hospitals, a tertiary teaching hospital and a central community hospital, providing approximately 630,000 and 150,000 patient consultations in 2019, respectively [15]. Six surgical wards were randomly chosen from a total of nine surgical wards and invited to participate, five at the teaching hospital and one at the central community hospital. All the six surgical wards accepted are as follows: ear, neck and throat (ENT)/maxillofacial, cardio-thoracic, neuro, breast and endocrine, gastrointestinal surgery and general surgery (central community hospital). The order to when the wards should start recruiting participants followed the randomised order of the planned larger SW-CRCT.

The data from this study were sampled from the same surgical patient population as the PASC development and initial validation study [10]. It was estimated a need to recruit 300 elective surgical patients, including 50 patients per cluster to investigate the content validation of the checklist [10] and the feasibility of the recruitment processes before the planned SW-CRCT. The estimation was based on the PASC trial protocol where a power calculation was performed to calculate the numbers of elective surgical patients needed per cluster in a larger SW-CRCT. Here, the initial numbers were 50 patients per cluster per month. All patients scheduled for elective surgery within the included specialties were prospectively invited to use both parts of PASC before surgery and discharge. Exclusion criteria were elective surgical patients aged < 18 years, not cognitively capable of reading or answering the checklist, living in supported accommodation (e.g. care or nursing homes) or not fluent in Norwegian. The patients received an invitation to use PASC within a period of 2 to 12 weeks before surgery; the exact timing depended on severity of disease and urgency of surgery.

### Data collection

Data were collected over a period of 14 months (August 2019 to end of September 2020) following a stepped-wedge recruitment design. The recruitment of participants and data collection were performed in cooperation with surgeons and nurses at each surgical ward. Both parts of PASC were collected before discharge from the patients who had consented to participate in the study. If participants did not deliver PASC at discharge, a single reminder was posted to them by mail, in which patients were asked to return the checklist/consent in an enclosed prepaid envelope.

Quantitative data collected included patient demographics including surgical ward, gender and age. Counts and percentages of the patient’s PASC item usage, return rate and any reasons for not returning the checklist were also collected. Four members (KH, ES, ASH and HVW) of the research team collected all data. The data quality was ensured by separate registrations and comparing the two registrations for errors. Any deviations were checked again.

For the qualitative part of the study, twenty-four postsurgical patients who had used both parts of PASC were invited to participate in three focus-group interviews 2- to 8-week post-surgery. The focus groups were carried out with patients invited from four surgical wards: ENT/maxillofacial, neuro, breast/endocrine and cardio-thoracic. The reason for only inviting patients from four of the six wards was the COVID-19 pandemic. In a period from March to end of May 2020, it was not possible to conduct focus-group interviews due to restrictions, and the patients from the remaining two wards were lost due to being more than 12 weeks since their surgery. All recruited participants had consented prior to surgery to be contacted by the research team regarding the focus-group interviews. All invited patients received a reminder the day before the interview. Out of the invited patients, 14 cancelled or did not attend. The topic guide used within the focus-groups interview was designed based on PASC items. All interviews were carried out by MR and KH, and a pilot interview was performed with the hospitals’ patient representatives. Each focus group interview lasted approximately 60 min and was carried out in a large meeting room, in accordance with the hospitals’ COVID-19 restrictions at time of data collection.

### Data analysis

Descriptive statistics were used to report on patient demographics, the return rate and the percentage of PASC item usage by patients. A chi-squared test was performed to investigate demographic gender differences between respondents and nonrespondents in this study. All statistical analysis were carried out in STATA version SE 16.1 (StataCorp. 2019, College Station, TX: StataCorp LLC). Focus group interviews with patients were audio recorded and transcribed verbatim for subsequent analysis. Text revealing barriers and drivers for PASC was collated into meaning units, which then were condensed, assigned a code and sorted into subcategories. The research team discussed the subcategories and reorganised them into categories. Content analyses were carried out and finalised at descriptive category level, following the procedure recommended by Graneheim and Lundman [16].

## Results

### Patient demographic information

A total of 428 patients were invited to use PASC during the recruitment period. Of those, 50.2% (215/428) consented to participate and returned completed checklists (Table 1). We found no significant gender difference between the patients who completed PASC and those who did not (*p* = 0.599). See Table 1 for description of differences in gender and ward between responders and nonresponders.

**Table 1:** Patients (*n* = 428) demographics and return rate of the patient safety checklist

Surgical specialties	Responder’s age (SD)	Return rate *n* (%)/females *n* (%)		Non-responders n (%)/Females n (%)	
Gastro	59.0 (12.8)	37 (45.7)	19 (51.4)	44 (54.3)	19 (43.2)
General	65.0 (14.4)	23 (38.9)	7 (31.2)	36 (61.0)	17(47.2)
Breast/endocrine	59.8 (9.8)	45 (72.6)	43 (95.6)	17 (30.9)	17(100)
ENT/maxillofacial	50.6 (15.3)	42 (50.6)	22 (52.4)	41 (49.4)	25(62.3)
Neuro	54.5 (9.6)	32 (48.5)	17 (15.1)	34 (51.5)	18(53.0)
Cardio-thoracic	62.7 (9.9)	36 (46.8)	6 (16.6)	41 (53.2)	12(29.5)
Total	58.0 (8.6)	215 (50.2)	114 (53.3)	213 (49.8)	108(49.5)

Abbreviations: *SD* Standard deviation, *ENT* Ear, neck and throat

### Checklist completion rate through the phases of the study and perioperative care

**Fig. 1:**
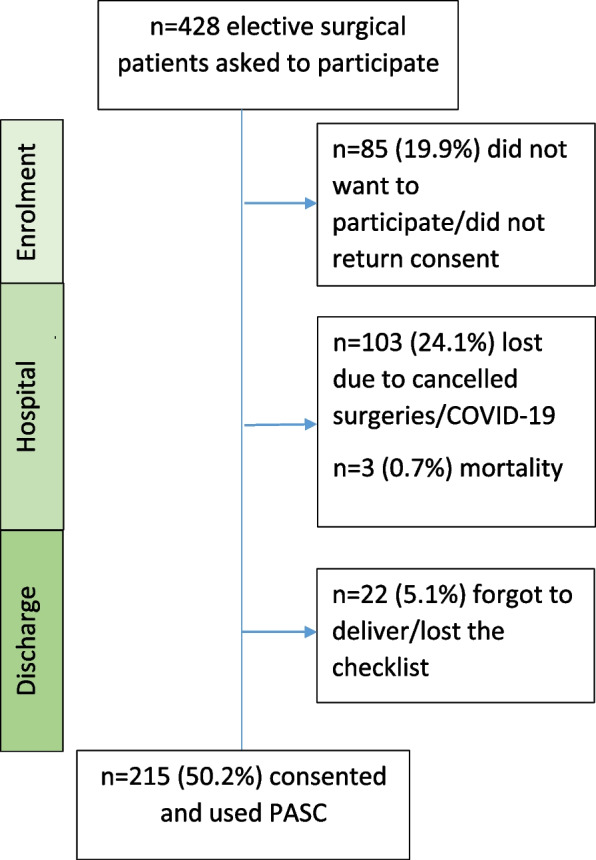
Flow chart of return rate of PASC and causes for nonresponders not returning PASC. A single reminder was sent out once to 9.8% (21/215) patients in the group who consented to use PASC and also to 50% (107/213) of the elective surgical patients who did not return the consent or forgot to deliver the checklist

Of the patients who did not consent, complete and/or return the checklist, 24.1% (103/428) were related to surgical or COVID-19-related cancellations, and 19.9% (85/428) did not consent to participate or did not reply at all to the study invitation. The last 5.1% (22/428) nonresponders lost the checklist. Finally, 0.7% (3/428) of the patients were lost to follow-up due to in-hospital mortality (Fig. 1).

### Fidelity of patients’ safety checklist completion

#### Overall PASC completion

The majority of patients who use PASC completed it; four out of five patients completed ≥ 80% of the items (86.5%, 186/215) (Table 2).

**Table 2:** PASC item total

Items completed %	Items completed (out of total of 56)	Patients *n* (%)
90–100	50–53	143 (66.5)
80–89	45–49	43 (20.0)
70–79	39–44	8 (7.2)
60–69	33–38	5 (2.3)
50–59	28–32	11 (5.1)
40–49	22–27	3 (1.4)
30–39	17–21	1 (0.5)
20–29	11–16	0 (0.0)
10–19	6–10	0 (0.0)
0–9	0–5	1 (0.5)
Total	56	215 (100)

#### Preoperative PASC item completion

A total of 96.7% (208/215) of patients who used preoperative PASC completed 80–100% of the items, 2.8% (6/215) completed 50–80% of the items and 0.47% (1/215) completed fewer than 50% of the 30 items on PASC before surgery (Table 3).

**Table 3:** Preoperative PASC items total

Items completed %	Items completed	Patients *n* (%)
90–100	27–30	188 (87.4)
80–89	24–26	20 (9.3)
70–79	21–23	3 (1.4)
60–69	18–20	1 (0.5)
50–59	15–17	2 (1.0)
40–49	12–14	0 (0.0)
30–39	9–11	0 (0.0)
20–29	6–8	0 (0.0)
10–19	3–5	0 (0.0)
0–9	0–2	1 (0.5)
Total	30	215 (100)

#### Postoperative PASC item completion

Table 4 shows that 73.0% (157/215) of the surgical patients completed 80–100% of the items, and that 17.5% (38/215) completed 50–79% of the items on the postoperative PASC. A total of 9.2% (20/215) of patients completed fewer than 50% of the items on the postoperative PASC (Table 4).

**Table 4:** Postoperative PASC items total

Items completed %	Items completed (out of total of 26)	Patients *n* (%)
90–100	24–26	117 (54.4)
80–89	21–23	40 (18.6)
70–79	18–20	21 (9.8)
60–69	16–17	13 (6.0)
50–59	13–15	4 (1.8)
40–49	10–12	4 (1.8)
30–39	8–9	0 (0.0)
20–29	5–7	0 (0.0)
10–19	3–4	0 (0.0)
0–9	0–2	16 (7.4)
Total	26	215 (100)

### Barriers and drivers to the use of the patients’ safety checklist: qualitative data

A total of ten patients were interviewed in three focus group interviews, four participants in two interviews and two participants in one interview (see Additional file 2 for full interview guide). Six of the patients were males and four women, with an age ranging from 37 to 64 years (mean age = 50 years, *SD* = 8.6 years).

Four categories were identified with several codes in each category, which represented the patients’ experiences of both barriers and drivers in using PASC — as follows: time frame for completing the checklist, patient safety checklist design, impetus to communication with healthcare professionals and support throughout the surgical pathway. See Additional file 3 for full analysis. In the following sections, the four categories are presented with illustrative patient quotes in italics.

#### Time frame for completing the checklist

The subcategories identified under this category represented barriers and drivers: the time of receiving preoperatively PASC and the time for completion of postoperative PASC. Some patients explained that when they received PASC before surgery, it was important that they were able and had time available to use it proactively. They further commented that PASC had to be received at the right time, not too early or too late before admission to the hospital in order to be able to follow the recommendations of the checklist:

"*When I received the checklist I had already been to the first consultation with the health professionals. So, I already had gotten answers on some parts of the checklist."*

"*The checklist before surgery, two weeks is a bit too late for some of the preparations."*

"*The Checklist should not be sent a month or two before, it should maybe be closer to your surgery date."*

Most of the patients found that the preoperative checklist was easier to use than the postoperative checklist. Some patients expressed a rushed discharge process as a barrier, not allowing enough time to use PASC or ask questions:

"*The checklist before discharge, you ended up doing after the doctor had seen you and discharged you. It went damn fast."*

"*There was not much time, you are laying there slightly lightheaded and suddenly you have to go home."*

However, not all patients found the postoperative PASC difficult to use:

"*I had no problems with the second part, just filled it out and delivered it."*

#### Patients’ safety checklist design

Patients’ experiences of barriers and drivers in relation to the subcategories under PASC design were identified as follows: some questions appearing rather too similar, availability of an electronic version, availability of an adjustable checklist and item understanding. One patient expressed that PASC had more items than necessary:

"*It had more questions than it needed to be."*

Several patients believed the checklist would be easier to use if it was delivered electronically. However, they did acknowledge that this would not be the case for all patients, and therefore, PASC should be made accessible as a paper version as well:

"*Paper rather than electronic checklist would simplify it."*

"*Some elderly like my mother, she would not be able to complete it, she and an app? You can forget it."*

Furthermore, when it came to understanding the PASC items, most of the patients stated that the items made good sense:

"*I understood the checklist because there was headings on each theme, so you could skip these or find the information you needed."*

"*The questions are written simple."*

Within this category, we also found that some patients found the postoperative PASC more complex, overall:

"*The first one (checklist) was not difficult."*

"*The checklist before discharge was more difficult to use."*

#### Impetus to communicate with healthcare professionals

Patients expressed that the main facilitator for utilising PASC was that it led them to asking for more information. PASC provided guidance of the surgical pathway and highlighted information that they otherwise would not have considered important:

"*There were a few questions that were not mentioned or I forgot to inform about it, I was experiencing a chaos and it was okay to have something that could help me, it is important that the hospital gets the information."*

"*I see there were very relevant questions for me, those with medications gave me an awakening."*

"*You get information about your situation. As an example I read that I had to inform the staff if I got cold in order to prevent infections and bleeding, I never knew that, so it was okay to get that information."*

Another driver for using PASC was that it encouraged the patients to ask healthcare professionals for important information:

"*Pain-relief (medication), I perceived that they almost forgot it and I would have not asked if I did not have the checklist."*

 "*I had to ask about marking of the operation side."*

 "*It was useful for me, helped me. I’m a person that asks a lot of questions during the consultations so it helped me to remember, it’s easy to forget."*

#### Support throughout the surgical pathway

All patients suggested that PASC gave an increased sense of safety and control by providing them with more systematised or structured information. However, they did express the need for more involvement from healthcare professionals. PASC created an increased focus on safety and control over their situation:

"*You create a focus around the situation that specifies it, you can go through it in a more systemised way, than you normally would do."*

"*Safety and maybe the checklist will improve the safety in the future."*

"*I felt it was good to have this, it helped me to gain control over things I had forgotten."*

Patients also commented that the checklist could be more effective if the healthcare professionals were more involved in the usage of PASC — especially the nurses. They believed that PASC should change the way they practice throughout the patient’s surgical pathway, and the health professionals should also use PASC as a guide to ensure that the patients had received and understood the information:

"*Ensure that both parts (patients and nurses) have understood the checklists’ purpose, a bit bureaucratic in the beginning, but over time they will get a better work routine than they have today."*

"*Ensure there are two checklists and then go through them together, then you are safe."*

"*The staff should use the checklist opposite way instead of only ask because it’s easy to say yes."*

## Discussion

Our quantitative findings show that elective surgical patients that receive PASC will consider using the checklist, and that four out of five patients who used PASC read/understood and completed the majority of its items (four out of five, overall). Qualitatively, PASC gave the patients an increased sense of safety, by guiding them through the surgical pathway and providing them with crucial information so that they could potentially prevent error or harm occurring to themselves. These findings are in line with current calls for research on how to involve patients in their own safety. Use of patient-completed checklists might be one of the keys for how to best empower patients’ involvement in safety within surgical and other medical fields [11, 12, 17].

We found that unplanned surgical cancellations inhibited patients from using PASC, and some dropped out of the study due to having lost it, failed to return the consent form or not completed PASC. These findings were expected prior to study start. The COVID-19 pandemic affected the study towards the end of the recruitment period due to all the elective surgeries that were cancelled in both hospitals during a COVID-19 peak period of 12 weeks. Even with this limitation, 50% of patients used the checklist. Elective surgery cancellations were not solely caused by COVID-19; they were also related to other known causes such as change of patient condition, lack of surgical staffing or facilities and lastly due to patients choosing to transfer to other hospitals, or not to have the operation/turn up [18]. Some of these causes for surgical cancellation can be prevented by improving patients’ preoperative evaluations and preparations, patient and healthcare professionals’ communications [19] — which reflects one of the purposes of having PASC in place. We hypothesise that PASC could help prevent patient-related surgical cancellations if most elective surgical patients used it.

Through qualitative data, we gathered a better understanding of barriers and drivers for PASC use, such as the importance of elective surgical patients receiving PASC at the right time, involvement of healthcare professionals in its usage and the importance of simplicity and good design. Importantly, our data showed that to achieve successful implementation and effect, PASC needs to be a tool mutually used by both patients and healthcare professionals. It appears that for PASC to reach its usability potential, it is important that the healthcare professionals go through both parts of the checklist together with the patients to ensure that the information and preparations are understood. This preliminary conclusion is supported by existing evidence, and that effective communications between patients and healthcare professionals are generally in medical care positively linked to patient experiences and quality of care [20–22]. Overall, these are early data, and the larger subsequent trial will further evaluate them.

Not all barriers related to PASC can be addressed easily, and the question of accessibility was raised by the patients in the focus group interviews of our study, as well as by patient representatives; this has also been raised by other researchers [17, 22, 23]. A checklist for patients might not be accessible to all patients, such as those with disabilities, visual impairment, learning difficulties, dementia, language barriers or multimorbidity in general. If a checklist of any type is only electronically available, further accessibility barriers might arise due to lack of IT access and/or literacy in some patients [4, 21, 23, 24]. Some patients may choose not to use the checklist because they have a more passive attitude to their care and find it difficult to become involved in conversations with healthcare professionals [21, 23]. It is important that such aspects of a patient-driven checklist implementation and uptake are carefully considered in further studies.

In terms of demographic factors impacting completion of the checklists, we did not find a difference between responders nonresponders in terms of gender, but we did find significant differences in response rates between study wards. The low response rate in the central community hospital may have been related to two causes: either the research team were not well represented during the patient recruitment period or that the central community hospital performed less complex surgeries compared with the university hospital. Relatedly, patients recruited in the central community hospital might also have been less sick and therefore busy with work and daily activities (i.e. more so than the patients recruited in the university hospital) and therefore had less time to use a checklist. In terms of research resources, more funding and research staff have been directed towards the central community hospital to eliminate this potential problem in light of the SW-CRCT to be launched after this feasibility study. Furthermore, the research team saw that the initial recruitment power calculation was not feasible. It was initially calculated to recruit 50 patients per surgical ward (total six surgical wards) a month over a period of 14 months. In preparation for the upcoming SW-CRCT, project funding has been increased, allowing for an increased number of clusters and recruitment period. In terms of the patient profile, further investigation is required to ensure good and balanced recruitment for the SW-CRCT across both teaching and non-teaching study sites.

### Limitations and strengths

The main limitation of the study is that data on outcomes as complications, mortality and hospitalisation time have not been evaluated. The methods to evaluate these outcomes have been thoroughly studied in former checklist studies in surgery within our research group [25, 26] and remain to be evaluated in the planned SW-CRCT. The feasibility evidence collected reflects the numbers of items used and the patients’ experiences of barriers and drivers to PASC use. The checklist was developed in a Norwegian context as a high-income country, known to have a culture that supports patient engagement [27]. Thus, it remains to be investigated how PASC will fit in with other nations’ healthcare systems and cultures. Another limitation is the low numbers of patients interviewed in the focus group interviews. This part of the study was carried out during the beginning of the COVID-19 pandemic, and several patients might have declined due to this. However, in the third focus group interview, we did not identify any new barriers or facilitators for using PASC that were not previously mentioned, which indicates at least some saturation of the data [28].

The PASC uptake rate at 50.2% (215/428) was satisfying, though likely influenced by the COVID-19 pandemic situation. A major strength in this study is the high percentage of checklist items completed by patients who engaged with PASC. In all, 86.5% (208/215) of the patients who used PASC filled in 80–100% of the checklist items. However, it has to be acknowledged that the data for this feasibility study was collected from the same patients in our previous development and validation study [10], and that the result for this study would have been strengthened if the data were collected separately.

## Conclusion

This study indicates that it is feasible for adult elective surgical patients to use the recently developed checklist, and that most patients are willing to use such a checklist. Patients reported that PASC can increase the communication between patients and healthcare professionals, to support and guide elective surgical patients through the tangles of information within the surgical pathway. PASC is feasible to use in a large-scale randomised controlled trial.

## Supplementary Information

**Additional file 1.** PASC item questions and response rate.

**Additional file 2.** Interview Guide.

**Additional file 3.** Coding tree focus group interviews.

**Additional file 4.** Data material.

## Data Availability

The dataset used and/or analysed during this study are available in English in Additional files 3 and 4. If more details are needed, please contact corresponding author on reasonable request.

## Abbreviations

WHO: World Health Organization
PASC: Patient’s safety checklist
SW-CRCT: Stepped-wedge cluster randomised controlled trial
COVID-19: Coronavirus disease 2019
CONSORT statement: Updated guidelines for reporting parallel group randomised trials
COREQ: Consolidated criteria for reporting qualitative research
ENT: Ear, neck and throat
SD: Standard deviation
