# Supplementary material for: Feasibility of implementing a surgical patient safety checklist: prospective cross-sectional evaluation
Source: Pilot Feasibility Stud. 2023 Mar 27;9:52. doi: 10.1186/s40814-023-01277-3 (PMC10040905; doi:10.1186/s40814-023-01277-3)
Supplement: Supplementary file 1 — Additional file 1. PASC item questions and response rate. [file 40814_2023_1277_MOESM1_ESM.docx]

| **PASC item questions and item response rate** | | | |
| --- | --- | --- | --- |
| **Preoperative PASC checklist items** | **Responders per item**  **n** | **Missing**  **per item**  **n** | **Missing**  **per item**  **%** |
| 1. Are you using any medications? | 214 | 1 | 0.5 |
| 2. Do you have a medication list with the latest changes? | 181 | 34 | 15.8 |
| 3. Are you using blood-thinning medications? | 211 | 4 | 2.0 |
| 4. Do you have diabetes, high blood pressure, cardio- vascular or receiving treatment for other chronic conditions? | 211 | 4 | 2.0 |
| 5. Do you have non-healing wounds? | 213 | 2 | 0.9 |
| 6. Have you received dental treatment, medical treatment, or been hospitalised, or worked in hospitals overseas the last 12 months? | 214 | 1 | 0.5 |
| 7. Are you informed that physical activity and a healthy diet before surgery can reduce chances for complications? | 211 | 4 | 2.0 |
| 8. Are you informed that stopping smoking, alcohol and substance abuse as early as possible before surgery can reduce chances of complications? | 206 | 9 | 4.2 |
| 9. Do you go to your dentist regularly? | 212 | 3 | 1.4 |
| 10. Have you read the admission letter and all other information given to you? | 206 | 9 | 4.2 |
| 11. Do you know what type of surgery you are having and the time of your surgery? | 213 | 2 | 0.9 |
| 12. Are you informed of when, or if you should stop your blood thinning medication before your surgery? | 181 | 34 | 15.8 |
| 13. Have you filled out all required forms before admission to the hospital? | 196 | 19 | 8.8 |
| 14. Do you have family or a close friend that can accompany you to get the information about your surgery? | 208 | 7 | 3.3 |
| 15. Are you under investigation for other diseases? | 209 | 6 | 2.8 |
| 16.Are you informed about what you should do if you get sick the week before surgery? | 211 | 4 | 2.0 |
| 17. Are you informed about how long you could expect to stay in hospital? | 209 | 6 | 2.8 |
| 18. Do you have family or a friend that can be with you the first night after you are discharged? | 210 | 5 | 2.3 |
| **Preoperative PASC checklist items** | **Responders per item**  **n** | **Missing**  **per item**  **n** | **Missing**  **per item**  **%** |
| 19. Ask if there are some things you need to have ready at home (bandages, medications) | 195 | 20 | 9.3 |
| 20. Do you need homecare or other social services? | 208 | 7 | 3.3 |
| 21. Are you informed if you need rehabilitation or physiotherapy? | 205 | 10 | 4.7 |
| 22. Have you removed all rings, necklaces, piercing, fake nails and nail polish? | 209 | 6 | 2.8 |
| 23. Are you informed about when you should stop eating and drinking before your surgery? | 209 | 6 | 2.8 |
| 24. Are you informed about hygiene/showering routines before your surgery? | 209 | 6 | 2.8 |
| 25. Do you have children under 18 years?. | 213 | 2 | 0.9 |
| 26. Are you allergic to any medication or medical equipment (latex)? | 214 | 1 | 0.5 |
| 27. Are you using any medications permanent or in periods | 212 | 3 | 1.4 |
| 28. Do you use any herbal medication or nutritional supplements? | 211 | 4 | 2.0 |
| 29. Are you informed about expected pain after your surgery? | 211 | 4 | 2.0 |
| 30. If relevant: have your operation site or side been marked? | 177 | 38 | 17.7 |
| 31. Avoid getting cold, inform your nurse | Not required a response from patient  (only a advice) | | |
| 32. Request the use of safe surgery checklist when you arrive at the surgical theatre. The surgical team should clarify your identity, type of operation and which side you should operate on (if you are having surgery on a side) | Not required a response from patient  (only a advice) | | |

| **Postoperative PASC Items** | **Responders per item**  **n** | **Missing**  **per item**  **n** | **Missing**  **per item**  **%** |
| --- | --- | --- | --- |
| 33. Are you informed about possible complications? | 196 | 19 | 8.8 |
| 34. Are you informed about what you should do if you experience complications or in an emergency? | 196 | 19 | 8.8 |
| 35. Are you informed if you need to take any special considerations after your surgery? | 192 | 23 | 10.7 |
| 36. Are you informed if you need compression stockings | 186 | 29 | 13.5 |
| 37. Are you informed about when you can drive after surgery? | 192 | 23 | 10.7 |
| 38. Are you informed about the importance of being physical active and when you can begin to exercise? | 190 | 25 | 11.6 |
| 39. Are you informed about activity restrictions? | 192 | 23 | 10.7 |
| 40. Are you informed about when you can shower again? | 188 | 27 | 12.6 |
| 41. Are you starting on new medications? | 196 | 19 | 8.8 |
| 42. Are you informed about possible side effects of your new medications? | 130 | 85 | 39.5 |
| 43. Are you informed about whom to contact if you are experiencing side effects? | 139 | 76 | 35.4 |
| 44. Are you informed about medications or food you cannot eat together with your new medications? | 130 | 85 | 39.5 |
| 45. Are you going to use your medication regularly? | 133 | 82 | 38.1 |
| 46. Have you stopped any medications (for example. blood thinners, blood pressure medications) in relation to your surgery? | 186 | 29 | 13.5 |
| 47. Have you received a copy of your new medication list? | 140 | 75 | 34.9 |
| 48. Do you need a prescription on pain relief medication? | 195 | 20 | 9.3 |
| 49. Are you informed about how to use and when you should stop taking pain relief? | 173 | 43 | 20.0 |
| 50. Are you informed about what you can do if recommended pain-relief dosage is not sufficient? | 173 | 50 | 23.3 |
| **Postoperative PASC Items** | **Responders per item**  **n** | **Missing**  **per item**  **n** | **Missing**  **per item**  **%** |
| 51. Are you informed that it can take some time before your bowel motions are back to normal and what to do for prevention? | 188 | 27 | 12.6 |
| 52. Are you informed about wound care, bandage changes, removal of sutures and who can help you with this? | 193 | 21 | 9.8 |
| 53. Are you going to have a follow up appointment? | 187 | 28 | 13.0 |
| 54. Are you referred to other medical specialities? | 187 | 28 | 13.0 |
| 55. Are you informed about whom you can contact after discharge if you have any questions or need to make enquire about follow-up appointments? | 191 | 24 | 11.2 |
| 56. Do you need a sick certificate? | 184 | 31 | 14.4 |
| 57. Are you informed whom to contact if you need a sick certificate extension? | 170 | 45 | 20.9 |
| 58. Are you informed about when you can travel after your operation? | 190 | 25 | 11.6 |

Colour coding as following based on previous development and validation study [1]:

| Item kept after the validation study |
| --- |
| Item reviewed and added to other items after the validation study |
| Item removed after the validation study |

1. Harris K, Søfteland E, Moi AL, Harthug S, Ravnøy M, Storesund A, et al. Development and validation of patients' surgical safety checklist. BMC Health Serv Res. 2022; Feb 25;22(1):259. <https://doi.org/10.1186/s12913-022-07470-z>
