# Supplementary material for: Feasibility of implementing a surgical patient safety checklist: prospective cross-sectional evaluation
Source: Pilot Feasibility Stud. 2023 Mar 27;9:52. doi: 10.1186/s40814-023-01277-3 (PMC10040905; doi:10.1186/s40814-023-01277-3)
Supplement: Supplementary file 3 — Additional file 3. Coding tree focus group interviews. [file 40814_2023_1277_MOESM3_ESM.docx]

| **CATEGORIES** | **SUB-CATEGORY** | **CODES** | **EXAMPLE** |
| --- | --- | --- | --- |
| Time frame for completing PASC | Point of receiving PASC | Received PASC after meeting with Healthcare professionals  Accelerated surgery time  Filled out PASC at home  Send out PASC closer to surgery  Need time to contact gp and to prepare  2 weeks before surgery is to late  PASC should be sent together with surgery date | *“When i received PASC i already had been to the first consultation with the health professionals. So I already had gotten answers on some parts of the checklist”.*  *“The surgery got suddenly a head of time”*  *“The Checklist should not be sent a month or two before it should maybe be closer to your surgery date”.*  *“ the checklist before I filled out at home”*  *“Two weeks before surgery, who have had time to address issues with their gp and prepared for surgery”.*  *“The checklist before surgery, two weeks is a bit to late to do preparations”.*    *" Maybe receive it a bit earlier before your operation date notice or at the same time"* |
|  | Completion time of postoperative PASC | To rushed discharge process  Little time to complete postop PASC  Did not have time to ask all the questions  Had to call the ward | *“The discharge process was to fast”*  *“ I used the checklist before, but the one after was rushed”*  *“The checklist before discharge you ended up doing after the doctor had seen you and discharged you. It went damn fast”*  *“ It was not much time, you are laying there slightly light headed and suddenly you have to go home”*  *“ I had no problems with the second part just filled it out and delivered it”*  *“I had a good discharge conversation but there was questions I should have asked from the list that I forgot. I had to call back to the hospital”* |
| **CATEGORIES** | **SUB-CATEGORY** | **CODES** | **EXAMPLE** |
| PASC design | To similar questions | Repetition of some questions  More questions than required | *“There was some questions that were the same”.*  *“Some questions were too similar”.*  *“More questions than it needed to be”.* |
|  | Electronic version | Able to access on computer  Not for all patient groups  Electronic version simplifies the checklist | *“Should be on a computer”.*  *“Some elderly like my mother, she would not be able to complete it, she and a app you can forget it”.*  *“Paper versus electronic checklist would simplify it”.* |
|  | Adjustable checklist | Don’t have to answer irrelevant questions  Jump items | *“If I crossed out no, I should be able to go to question 7 instead of number 3”.*  *“If you answered yes or no you could jump next question if it was irrelevant”..* |
|  | Understanding the items | First page was easy to understand  Need better information about optimising own health  Items are easy to understand  Part two was more difficult to understand  Misunderstood meaning of one question  Headings made it easy to navigate | *“Page one is alright, it is only general information questions”.*  *“Are you going to have a surgery you should have good health, if your gp see it’s going towards surgery. The information about you lifestyle is not good enough”.*  *“The questions are written simply”.*  *“The checklist before discharge was more difficult to answere”.*  *“The first one was not difficult”.*  *“The first part were you should fill out at home was easier than the one before discharge”.*  *«This question about stomach functions I miss understood it. I’m not having an abdominal surgery and then i got constipated and understood the meaning”.*  *“I understood the checklist because there was headings on each theme, so you could jump over or find the information you needed”.* |
| **CATEGORIES** | **SUB-CATEGORY** | **CODES** | ***EXAMPLE*** |
| Impetus to communication with health professionals | Utility value of PASC | Gave important information  Repetition of information led to feeling of safeness  Most questions were covered  Used advise in PASC to check own medications | “There was a few questions that was not mentioned or I forgot to inform about it, I was experiencing a chaos and it was okey to have something that could help me, it is important that the hospital gets the information”.  “I felt well cared of, there was a lot of the same questions, but at the same time we should not experiences any errors”.  “It gave reminders of what needed to be done before surgery, especially this with the dentist”.  “The information about what you could do and not and about complications was covered by the nurse”.  “There was information that was god to get such as this with fever or infections, then you had to contact them straight away”.  “information about acute bleeding or fever than you had to come in straight away”.  “When I received my medications I checked them”.  “ I see there was very relevant questions for me, this with medications gave me a awakening”.  "you get information about your situation, as an example I read that I had to inform the staff if I got cold, I have never knew that so it was okay to get that information." |
|  | PASC led patients to ask for information | PASC information led to questions  Used the checklist to ask questions | “It was a side-effect that I did not get informed about, but I found it in the checklist”.  “I asked for information about training”.  “Pain-relief I perceived that they almost forgot it and I would have not asked if I did not have the checklist”.  “I read through the list to check if there was anything important or I had questions”.  “Many of the questions I wanted to ask about was covered, I did not have to ask most of the questions”.  “I had to ask about marking the operation side”.  “I had a question from the list I had to ask”  “It was useful for me, helped me. I’m that person that askes allot of questions during the consultations so it helped me to remember, it’s easy to forget”. |
| **CATEGORIES** | **SUB-CATEGORY** | **CODES** | EXAMPLE |
| Support throughout the surgical pathway | Increased systemising | Gained control  Created focus  Better cared for  Safety  Better information | “I felt it was good to have this, it helped me to gain control over things I had forgotten”.  “You create a focus around the situation that concretizes it you can go through it in a more systemised way, than you normally would do”.  “I felt better cared for in a way, its good that people trie to improve the system”.  “Safety and maybe the checklist will improve the safety in the future, because we use it”.  altså de de systematiserer, det med å bli systematisk i forhold til disse tingene og ha fokus på  «It was okay to go through the questions. Some things i did not now”.  “The checklist lead to better focus”. |
|  | Involve healthcare proffesionals | Clearefy questions  Ask if information is understood  Ask opposite way  Everyone should use PASC including healthcare staff. | “In the ward that the staff ensure that we have enough time to go through the checklist and ask if we have control or if there is anything that is unclear”.  “The staff should come and ask the opposite way instead of only ask because it’s easy to say yes”.  “ask if you have understood the questions”.  “Ensure there is two checklists and then go though it together, then you are safe”.  “Ensure that both parts have understood the checklists purpose, a bit bureaucratic in the beginning, but over time they will get a better work routine than they have today”.  “If they ask at the same time as you are reading through will increase your understanding”. |
