# Supplementary material for: Feasibility of implementing a surgical patient safety checklist: prospective cross-sectional evaluation
Source: Pilot Feasibility Stud. 2023 Mar 27;9:52. doi: 10.1186/s40814-023-01277-3 (PMC10040905; doi:10.1186/s40814-023-01277-3)
Supplement: Supplementary file 4 — Additional file 4. Data material. [file 40814_2023_1277_MOESM4_ESM.docx]

| Patient (n) | Missing items out of total (56) | Percent Missing |
| --- | --- | --- |
| 1 | 13 | 23.21 |
| 2 | 10 | 17.86 |
| 3 | 0 | 0.00 |
| 4 | 0 | 0.00 |
| 5 | 1 | 1.79 |
| 6 | 0 | 0.00 |
| 7 | 0 | 0.00 |
| 8 | 0 | 0.00 |
| 9 | 2 | 3.57 |
| 10 | 0 | 0.00 |
| 11 | 4 | 7.14 |
| 12 | 0 | 0.00 |
| 13 | 7 | 12.50 |
| 14 | 4 | 7.14 |
| 15 | 1 | 1.79 |
| 16 | 8 | 14.29 |
| 17 | 0 | 0.00 |
| 18 | 5 | 8.93 |
| 19 | 2 | 3.57 |
| 20 | 0 | 0.00 |
| 21 | 5 | 8.93 |
| 22 | 1 | 1.79 |
| 23 | 0 | 0.00 |
| 24 | 4 | 7.14 |
| 25 | 0 | 0.00 |
| 26 | 8 | 14.29 |
| 27 | 5 | 8.93 |
| 28 | 27 | 48.21 |
| 29 | 0 | 0.00 |
| 30 | 1 | 1.79 |
| 31 | 0 | 0.00 |
| 32 | 9 | 16.07 |
| 33 | 0 | 0.00 |
| 34 | 2 | 3.57 |
| 35 | 0 | 0.00 |
| 36 | 3 | 5.36 |
| 37 | 0 | 0.00 |
| 38 | 0 | 0.00 |
| 39 | 10 | 17.86 |
| 40 | 26 | 46.43 |
| 41 | 1 | 1.79 |
| 42 | 0 | 0.00 |
| 43 | 0 | 0.00 |
| 44 | 0 | 0.00 |
| 45 | 1 | 1.79 |
| 46 | 9 | 16.07 |
| 47 | 1 | 1.79 |
| 48 | 39 | 69.64 |
| 49 | 5 | 8.93 |
| 50 | 0 | 0.00 |
| 51 | 31 | 55.36 |
| 52 | 0 | 0.00 |
| 53 | 0 | 0.00 |
| 54 | 8 | 14.29 |
| 55 | 1 | 1.79 |
| 56 | 0 | 0.00 |
| 57 | 0 | 0.00 |
| 58 | 5 | 8.93 |
| 59 | 2 | 3.57 |
| 60 | 0 | 0.00 |
| 61 | 19 | 33.93 |
| 62 | 0 | 0.00 |
| 63 | 0 | 0.00 |
| 64 | 1 | 1.79 |
| 65 | 8 | 14.29 |
| 66 | 29 | 51.79 |
| 67 | 6 | 10.71 |
| 68 | 4 | 7.14 |
| 69 | 7 | 12.50 |
| 70 | 9 | 16.07 |
| 71 | 6 | 10.71 |
| 72 | 6 | 10.71 |
| 73 | 11 | 19.64 |
| 74 | 0 | 0.00 |
| 75 | 5 | 8.93 |
| 76 | 0 | 0.00 |
| 77 | 1 | 1.79 |
| 78 | 10 | 17.86 |
| 79 | 0 | 0.00 |
| 80 | 0 | 0.00 |
| 81 | 3 | 5.36 |
| 82 | 5 | 8.93 |
| 83 | 2 | 3.57 |
| 84 | 3 | 5.36 |
| 85 | 10 | 17.86 |
| 86 | 7 | 12.50 |
| 87 | 5 | 8.93 |
| 88 | 6 | 10.71 |
| 89 | 3 | 5.36 |
| 90 | 1 | 1.79 |
| 91 | 32 | 57.14 |
| 92 | 5 | 8.93 |
| 93 | 0 | 0.00 |
| 94 | 0 | 0.00 |
| 95 | 1 | 1.79 |
| 96 | 9 | 16.07 |
| 97 | 1 | 1.79 |
| 98 | 12 | 21.43 |
| 99 | 8 | 14.29 |
| 100 | 14 | 25.0 |
| 101 | 56 | 100.00 |
| 102 | 0 | 0.00 |
| 103 | 15 | 26.79 |
| 104 | 8 | 14.29 |
| 105 | 4 | 7.14 |
| 106 | 1 | 1.79 |
| 107 | 1 | 1.79 |
| 108 | 3 | 5.36 |
| 109 | 4 | 7.14 |
| 110 | 1 | 1.79 |
| 111 | 0 | 0.00 |
| 112 | 2 | 3.57 |
| 113 | 5 | 8.93 |
| 114 | 6 | 10.71 |
| 115 | 0 | 0.00 |
| 116 | 0 | 0.00 |
| 117 | 0 | 0.00 |
| 118 | 26 | 46.43 |
| 119 | 7 | 12.50 |
| 120 | 5 | 8.93 |
| 121 | 2 | 3.57 |
| 122 | 0 | 0.00 |
| 123 | 0 | 0.00 |
| 124 | 7 | 12.50 |
| 125 | 1 | 1.79 |
| 126 | 0 | 0.00 |
| 127 | 7 | 12.50 |
| 128 | 0 | 0.00 |
| 129 | 5 | 8.93 |
| 130 | 7 | 12.50 |
| 131 | 7 | 12.50 |
| 132 | 1 | 1.79 |
| 133 | 2 | 3.57 |
| 134 | 0 | 0.00 |
| 135 | 3 | 5.36 |
| 136 | 5 | 8.93 |
| 137 | 5 | 8.93 |
| 138 | 9 | 16.07 |
| 139 | 14 | 25.00 |
| 140 | 13 | 23.21 |
| 141 | 0 | 0.00 |
| 142 | 0 | 0.00 |
| 143 | 8 | 14.29 |
| 144 | 0 | 0.00 |
| 145 | 0 | 0.00 |
| 146 | 28 | 50.00 |
| 147 | 10 | 17.86 |
| 148 | 0 | 0.00 |
| 149 | 3 | 5.36 |
| 150 | 3 | 5.36 |
| 151 | 1 | 1.79 |
| 152 | 26 | 46.43 |
| 153 | 18 | 32.14 |
| 154 | 0 | 0.00 |
| 155 | 3 | 5.36 |
| 156 | 18 | 32.14 |
| 157 | 6 | 10.71 |
| 158 | 0 | 0.00 |
| 159 | 20 | 35.71 |
| 160 | 2 | 3.57 |
| 161 | 7 | 12.50 |
| 162 | 12 | 21.43 |
| 163 | 27 | 48.21 |
| 164 | 1 | 1.79 |
| 165 | 0 | 0.00 |
| 166 | 11 | 19.64 |
| 167 | 5 | 8.93 |
| 168 | 3 | 5.36 |
| 169 | 5 | 8.93 |
| 170 | 6 | 10.71 |
| 171 | 0 | 0.00 |
| 172 | 8 | 14.29 |
| 173 | 7 | 12.50 |
| 174 | 1 | 1.79 |
| 175 | 0 | 0.00 |
| 176 | 4 | 7.14 |
| 177 | 26 | 46.43 |
| 178 | 7 | 12.50 |
| 179 | 3 | 5.36 |
| 180 | 7 | 12.50 |
| 181 | 0 | 0.00 |
| 182 | 0 | 0.00 |
| 183 | 5 | 8.93 |
| 184 | 6 | 10.71 |
| 185 | 8 | 14.29 |
| 186 | 13 | 23.21 |
| 187 | 3 | 5.36 |
| 188 | 4 | 7.14 |
| 189 | 1 | 1.79 |
| 190 | 0 | 0.00 |
| 191 | 4 | 7.14 |
| 192 | 3 | 5.36 |
| 193 | 6 | 10.71 |
| 194 | 1 | 1.79 |
| 195 | 0 | 0.00 |
| 196 | 0 | 0.00 |
| 197 | 2 | 3.57 |
| 198 | 0 | 0.00 |
| 199 | 4 | 7.14 |
| 200 | 26 | 46.43 |
| 201 | 0 | 0.00 |
| 202 | 2 | 3.57 |
| 203 | 2 | 3.57 |
| 204 | 1 | 1.79 |
| 205 | 6 | 10.71 |
| 206 | 5 | 8.93 |
| 207 | 1 | 1.79 |
| 208 | 28 | 50.00 |
| 209 | 0 | 0.00 |
| 210 | 0 | 0.00 |
| 211 | 1 | 1.79 |
| 212 | 0 | 0.00 |
| 213 | 28 | 50.00 |
| 214 | 26 | 46.43 |
| 215 | 18 | 32.14 |
